# Multistage entanglement swapping using superconducting qubits in the absence and presence of dissipative environment without Bell state measurement

**DOI:** 10.1038/s41598-023-43592-y

**Published:** 2023-09-28

**Authors:** S. Salimian, M. K. Tavassoly, M. Ghasemi

**Affiliations:** Laser and Optics Group, Faculty of Physics, Yazd University, Yazd, Iran

**Keywords:** Quantum optics, Quantum information

## Abstract

In recent decades the entangled state generation is of great importance in the quantum information processing and technologies. In this paper, producing the distributed entangled state of superconducting (SC) qubits is considered using an entanglement swapping protocol in three successive stages. The SC qubit pairs $(i,\,i+1$ with $i=1,\,3,\,5,\,7)$, where each pair of the qubits has been placed on a separate chip, are initially prepared in maximally entangled states. The external magnetic fields on capacitively coupled pairs $(2,\,3)$ and $(6,\,7)$ are implemented for modulating the frequency of qubits. Then, the SC qubits $(1,\,4)$ and $(5,\,8)$ are converted into entangled states via operating proper measurements instead of Bell state measurement (which is generally a hard task). Finally, the distributed entangled state of target SC qubits $(1,\,8)$ can be obtained by applying external magnetic fields on qubits $(4,\,5)$ and via operating suitable measurements. This process is studied in the absence and presence of thermal decoherence effects. The concurrence, as a measure of entanglement between two target qubits, success probability of the distributed entangled states and the corresponding fidelities are evaluated, by which we find that the state of target SC qubits $(1,\,8)$ is converted to Bell state with maximum entanglement at some moments of time. Under appropriate conditions the maximum of success probability of the obtained states in each stage approaches 1. However, the maxima of concurrence and success probability gradually decrease due to the thermal noise as time goes on. Moreover, compelling amounts of fidelity, success probability and entanglement can be obtained for the achieved entangled states.

## Introduction

Quantum systems that have never interacted can be entangled through an entanglement swapping protocol^1–6^. The protocol of entanglement swapping is an essential tool for quantum communication^7^. The key role of quantum entanglement and quantum teleportation in reaching the quantum internet is undeniable. The entanglement swapping is a significant core of quantum repeater^8,9^ and quantum internet^10^.

Distribution of entanglement and entangled states plays an important role in quantum information processing.

It should be stated that, although entanglement swapping started a few years ago, it is still of noticeable importance and new works are being published^11–18^. Recently, entanglement swapping in the presence of dissipation and Kerr medium has been studied^19,20^. Entanglement generation between SC qubits which are not connected is essential in quantum computers, because quantum algorithms require coupling between qubits^21,22^. A scheme for the stationary generation of two distinct classes of entangled states, i.e. Werner-like and maximally entangled mixed states in an open quantum system has been recently proposed by one of us^23^. Also, entanglement swapping is performed by beam splitter^24^, Mach-Zehnder interferometer^25^ and cavity QED^26,27^. In^28^, entanglement swapping between SC qubits has been considered to design a quantum repeater protocol.

**Figure 1:**
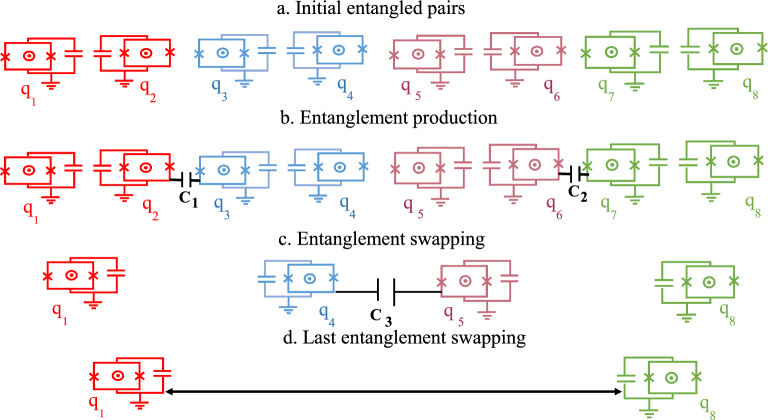
The scheme of entanglement swapping protocol. At first, four entangled transmon qubit pairs $(1,\,2)$, $(3,\,4)$, $(5,\,6)$ and $(7,\,8)$ have been considered such that each pairs of the entangled qubits has been placed on a separate chip. The transmon qubits $(2,\,3)$, $(6,\,7)$ and then $(4,\,5)$ are capacitively coupled to each other respectively via capacitance inductance $C_i$ $(i=1,\,2,\,3)$. The tunable interaction between each of the two qubits can be realized by varying the frequency of the external magnetic field, through the *j*th qubit $(j=3,\,5,\,7)$. Finally, the target qubits (1, 8) are entangled.

In the present study, due to the importance of SC circuits and qubits in quantum information processes^29,30^, the entanglement swapping protocol is designed using SC qubits which are based on Josephson junction. In fact, in SC qubits the nonlinearity arisen from Josephson junctions results in the nonuniform energy-level separation. This property allows one to encode a qubit in the lowest two levels of a SC circuit for implementing quantum computing and simulation^31^. These solid-state qubits, i.e., SC qubits, can be controlled by applied bias current, gate voltage, and microwave fields^31^. Also, SC qubits can strongly couple to each other by electromagnetic fields, where this strong coupling leads to short coherence times. So, improving the coherence properties is also one of the paramount priorities of the SC qubits^31^. The SC qubits which are at macroscopic scales can act as the artificial atoms. In addition, because of the strong coupling of SC qubits with electromagnetic fields (compared to natural atoms), they are more appropriate for quantum information processing phenomena on a chip^32,33^. In^34^, an experimentally realizable method to control the coupling between two flux qubits is presented. Recently, a quantum switch scheme has been implemented for coupling SC qubits connected by a gap-tunable bridge qubit where the two initial separated SC qubits are entangled by modulating the frequency of bridge qubit^35^. A hybrid superconductor-optical quantum repeater is provided in^36^. Controllability of coupling strength between SC qubits with each other via applying the external magnetic field is the other advantage of SC qubits^37–42^. Also, quantum networks and quantum repeater protocols require memories to save and release the entangled states. The performance of the storage and retrieval of quantum memories is improved using SC quantum processors and solid-state quantum memories^43^. The above-mentioned advantages and interesting properties of SC motivated us to consider the distribution of entangled states of target SC qubits $(1,\,8)$ among SC qubits $(1,2,\ldots ,8)$ which are aligned as in Fig. 1. The SC pairs $(i,\,i+1)$ where $i=1,\,3,\,5,\,7$ are initially prepared in maximally entangled states^44,45^. The entangled states for SC qubits $(1,\,4)$ and $(5,\,8)$ are achieved by implementing external magnetic fields on capacitively coupled pairs $(2,\,3)$ and $(6,\,7)$ followed by operating proper measurements. Then, the distributed entangled state of target SC qubits $(1,\,8)$ can be obtained by applying external magnetic fields on qubits $(4,\,5)$ and operating suitable measurements. To study entanglement swapping protocol in real conditions the effect of dissipation is also considered via the thermal noise influences. To explain about the possible experimental implementation of our proposal in the present work and its feasibility, we should refer to the literature in which some experimental setups, more or less like the one we modeled here, have been recently proposed (see Refs.^46–49^). In more detail, for instance, flux-tunable SC transmon for quantum information processing purposes is presented experimentally in^49^. Also, a chain of transmon qubits is connected capacitively to each other and quantum state transfer is studied experimentally in^46^. Although entanglement swapping between SC circuits has been recently considered in^50^, however, in comparison, our present work possesses essential characterizations. For instance, the authors used coupling of SC qubits to a resonator, but in our work, SC qubits are capacitively connected to each other. Secondly, the authors implemented the Bell state measurement, while it is a well-known fact that the Bell state measurement is not generally a straightforward process in quantum measurements. In fact, the realization and discernment of the four Bell states in experiment that is needed in the Bell state measurement method is still practically difficult^51–53^. Keeping in mind the mentioned fact, we have never used the Bell state measurement in this paper.

In relation to this point, it should be emphasized that, entanglement swapping without Bell state measurement method is of enough interest to the people who work in this field^54–56^. After all, even though the system setup and techniques used for the entanglement swapping in our paper and Ref.^50^ are essentially different, this Ref. can be confirmed that our proposal for entanglement swapping using the SC qubits is not far from the experimental realization.

This paper is organized as follows: decoherence-free entanglement swapping protocol to distribute entangled state of SC qubits is introduced and discussed in “Decoherence-free entanglement swapping based on SC qubits” section. Then, the protocol is considered in the presence of dissipation in “Dissipative entanglement swapping based on SC qubits” section. Finally, the paper ends with a summary and conclusions in “Summary and conclusions” section.

## Decoherence-free entanglement swapping based on SC qubits

In this section we want to introduce our multistage entanglement swapping protocol. Initially, as shown in Fig. 1, we assume that four SC qubit pairs (i; i + 1), where i = 1, 3, 5, 7, have been prepared in maximally entangled states. It should be mentioned that each pairs of the entangled qubits has been placed on a separate chip. Then, by implementing external magnetic fields on capacitively coupled pairs $(2,\,3)$ and $(6,\,7)$ and operating proper measurements instead of Bell state measurement the SC qubits $(1,\,4)$ and $(5,\,8)$ are converted into entangled states. Finally, the entangled state of target SC qubits $(1,\,8)$ is achieved by applying external magnetic fields on qubits $(4,\,5)$ and operating suitable measurements.

In this work, the crosstalk interaction is plenty suppressed via considering high detuning SC qubits^35^. Furthermore, the Stark shift effect on qubits does not need to be considered^46,49,57,58^.

In this way, four SC qubits $(1,\,2,\,3,\,4)$ are considered where the initial state of them reads as $|\psi \rangle _{1,2}\otimes |\psi \rangle _{3,4}$, each is defined as,

1 $$\begin{aligned} |\psi \rangle _{i,i+1}=\dfrac{1}{\sqrt{2}}\left( |e,g\rangle +|g,e\rangle \right) _{i,i+1},\,\,\ i=1,\,3. \end{aligned}$$

In this regard, we recall that in Refs.^59–61^ different schemes have been investigated for generating high fidelity entanglement between two distant SC qubits. In addition, another way for generating maximally entangled state of qubits is coupling two qubits with one SQUID or a resonator^35,62^ or by applying different quantum gates^63^. These entangled SC qubits can even be far apart, while maintaining their quantum states^64^. It should be mentioned that, the detuning between transmon qubits is sufficiently large, so the initial interaction between qubits can be neglected. In transmon qubits, the controllable Josephson energy ($E_J=E_{J,max} \left| \cos (\pi \Phi /\Phi _0)\right|$) via the magnetic flux $\Phi$ is more larger than the charge energy ($E_C=\dfrac{e^2}{2C}$); where *C* is either the capacitance of a Josephson junction or an island, depending on the circuit and $\Phi _0$ is the flux quantum^31^. The transition frequency between the first excited state and the ground state of the transmon qubits, i.e., $\omega \approx \sqrt{8E_J E_C}/\hbar$, can be tuned using the applied magnetic flux. In the following, the external ac magnetic field is applied to qubit 3 to modulate its frequency periodically with $\omega _3 = \omega _{03} + \epsilon _3 \sin (\nu _3t)$; where $\omega _{03}$ is the mean operating frequency. Also, $\epsilon _3$ and $\nu _3$ are the amplitude modulation and frequency of the external magnetic field, respectively. This type of modulation can be found in recent works^46,48,49^ where an external field has been used to modulate the frequency of the transmon qubit, as we considered here. We assume that $\omega _2=\omega _{02}$ since the qubit 2 is not modulated. Modulation of the frequency of qubits acts as a switch in this protocol^46^, i.e., if the frequency modulation is interrupted, the protocol will not be performed. Now, the interaction is proceeded between two non-entangled SC qubits $(2,\,3)$ where this interaction is described by the following Hamiltonian^46^,

2 $$\begin{aligned} H=\sum _{j=2}^{3}\dfrac{\omega _j}{2}\sigma _{j}^{z}+G\left( \sigma _{3}^{+}+\sigma _{3}^{-} \right) \left( \sigma _{2}^{+}+\sigma _{2}^{-} \right) . \end{aligned}$$

In Hamiltonian (2), $\omega _j$ denotes the frequency of *j*th transmon qubit, $\sigma _{j}^{z}$ ($\sigma _{j}^{\pm }$) is Pauli (ladder) operator of *j*th transmon qubit, and *G* is the coupling strength of the transmon qubits (2, 3). Two unitary operators are defined as,

3 $$\begin{aligned} U_1= \exp \left( -i\sum _{j=2}^{3}\dfrac{\omega _{0j}}{2} \sigma _j^z t\right) ,\qquad \qquad U_2= \exp \left( i \sigma _3^z \dfrac{\alpha _3}{2}\cos (\nu _3 t)\right) , \end{aligned}$$

where $\alpha _3=\varepsilon _3/\nu _3$. The transformed Hamiltonian may be obtained by applying the rotating frame $U=U_1\times U_2$ on Hamiltonian (2) as $H_I= U^\dagger H U+i \dfrac{d U^{\dagger }}{dt}U$^65,66^, which results in,

4 $$\begin{aligned} H_I &= G \left( \sigma ^+_2\sigma _3^-e^{-i\Delta t} \exp \left[ i \alpha _3 \cos (\nu _3 t)\right] + H.c.\right) , \end{aligned}$$

where $\Delta =\omega _{03}-\omega _{02}$. Using the Jacobi-Anger expansion as

5 $$\begin{aligned} \exp \left[ i\alpha \cos (\nu t) \right] =\sum _{m=-\infty }^{\infty } i^m J_m (\alpha )\exp \left( im\nu t\right) , \end{aligned}$$

and setting $\Delta =\nu _3$ which denotes to the sideband excitation process^49^, and also neglecting the fast oscillating terms under RWA (rotating wave approximation) which is a commonly used approximation technique in quantum physics and quantum optics^67–70^, the effective tunable Hamiltonian is achieved as below,

6 $$\begin{aligned} H_{\textrm{eff}}=\lambda \left( \sigma _2^+ \sigma _3^-+\sigma _2^- \sigma _3^+\right) , \end{aligned}$$

where the effective coupling strength is introduced as $\lambda = G J_1(\alpha _3)$ with $J_1(\alpha _3)$ as the first order Bessel function of the first kind. Notice that the fast oscillation terms in Eq. (4) have been neglected via RWA under the condition $\omega _j\gg G$. Using the effective Hamiltonian (6), the initial state $|\psi \rangle _{1,2}\otimes |\psi \rangle _{3,4}$ with the definition introduced in (1) and the time-dependent Schrödinger equation, the entangled state of SC qubits (1-4) can be achieved as follows:

7 $$\begin{aligned} |\psi (t)\rangle _{1-4} &=  \left( L_2(t) |e,g\rangle _{1,4}+ L_3(t) |g,e\rangle _{1,4}\right) |e,g\rangle _{2,3} +\left( L_1(t) |e,g\rangle _{1,4}+L_4(t) |g,e\rangle _{1,4}\right) |g,e\rangle _{2,3}\nonumber \\{} & {} + L_5(t) |e,e\rangle _{1,4}|g,g\rangle _{2,3}+L_6(t) |g,g\rangle _{1,4}|e,e\rangle _{2,3}, \end{aligned}$$

where

8 $$\begin{aligned} L_1(t) &=  L_3(t)=-\dfrac{i}{2}\sin (\lambda t),\nonumber \\ L_2(t) &=  L_4(t)=\dfrac{1}{2}\cos (\lambda t),\qquad \qquad L_5(t)= L_6(t)=\dfrac{1}{2}. \end{aligned}$$

Now, by applying two measurements $|e,g\rangle _{2,3}$ and $|g,e\rangle _{2,3}$ performed on the state obtained in (7), the entangled states of SC qubits $(1,\,4)$ are respectively achieved as follow (to make such measurement, each qubit should be individually connected to the LC resonator, not shown in Fig. 1),

9 $$\begin{aligned} |\psi (t)\rangle _{1,4} &=  \dfrac{1}{\sqrt{P_{1,4}(t)}}\left( L_2(t)|e,g\rangle +L_3(t)|g,e\rangle \right) _{1,4},\nonumber \\ |\psi ^{\prime }(t)\rangle _{1,4} &=  \dfrac{1}{\sqrt{P^{\prime }_{1,4}(t)}}\left( L_1(t)|e,g\rangle +L_4(t)|g,e\rangle \right) _{1,4}, \end{aligned}$$

where

10 $$\begin{aligned} P_{1,4}(t)= |L_2(t)|^2+|L_3(t)|^2,\qquad \qquad \qquad P^{\prime }_{1,4}(t)= |L_1(t)|^2+|L_4(t)|^2, \end{aligned}$$

are the success probabilities of the produced entangled states in (9). Also, the degrees of entanglement of states in (9) are respectively calculated via concurrence^71^ result in,

11 $$\begin{aligned} C(t)=\dfrac{2|L_2^{*}(t) L_3(t)|}{P_{1,4}(t)},\qquad \qquad \qquad C^{\prime }(t)=\dfrac{2|L_1^{*}(t) L_4(t)|}{P_{1,4}^{\prime }(t)}. \end{aligned}$$

Even though the generation of the entanglement between SC qubits (1, 4) can also be obtained by implementing universal gates (see Appendix A). All of these processes can be easily repeated for the qubits (5–8). That is, the interaction introduced in Eq. (6) can be performed between SC qubits $(6,\,7)$ and the entangled state (7) is obtained for the four SC qubits $(5,\,6,\,7,\,8)$. In the continuation, by operating two measurements $|e,g\rangle _{6,7}$ or $|g,e\rangle _{6,7}$ performed with qubits $(6,\,7)$ on state (7) related to SC qubits (5-8), the entangled states of SC qubits $(5,\,8)$ are produced as

12 $$\begin{aligned} |\psi (t)\rangle _{5,8}=|\psi (t)\rangle _{1,4},\qquad \qquad |\psi ^{\prime }(t)\rangle _{5,8}=|\psi ^{\prime }(t)\rangle _{1,4}, \end{aligned}$$

where $|\psi (t)\rangle _{1,4}$ and $|\psi ^{\prime }(t)\rangle _{1,4}$ have been defined in (9). Now, there exist four initial states for SC qubits $(1,\,4,\,5,\,8)$, i.e., $|\psi (t)\rangle _{1,4}\otimes |\psi (t)\rangle _{5,8}$, $|\psi (t)\rangle _{1,4}\otimes |\psi ^{\prime }(t)\rangle _{5,8}$, $|\psi ^{\prime }(t)\rangle _{1,4}\otimes |\psi (t)\rangle _{5,8}$ and $|\psi ^{\prime }(t)\rangle _{1,4}\otimes |\psi ^{\prime }(t)\rangle _{5,8}$, where the final interaction is performed at time $\tau$ ($\tau > t$) between the SC qubits $(4,\,5)$. For simplicity, the mentioned initial states for qubits $(1,\,4,\,5,\,8)$ are respectively shown by a general state with subscripts $i={1,\,2,\,3,\,4}$ as,

13 $$\begin{aligned} |\psi _{i}(t)\rangle _{1,4,5,8}=\left( \zeta _i(t)|e,g,e,g\rangle +\beta _i(t)|e,g,g,e\rangle \right. +\left. \xi _i(t)|g,e,e,g\rangle +\eta _i(t)|g,e,g,e\rangle \right) _{1,4,5,8}. \end{aligned}$$

**Figure 2:**
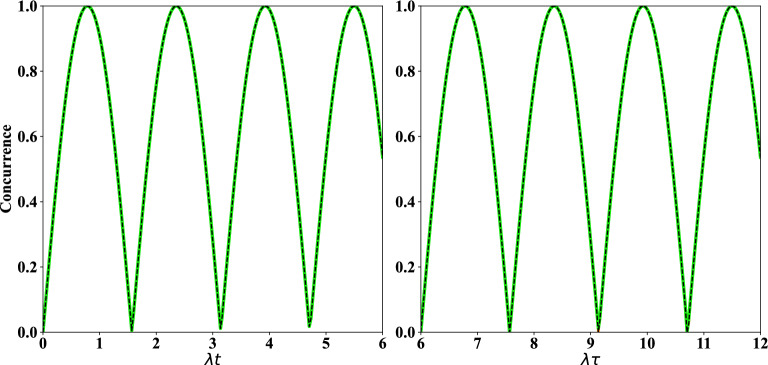
Left plot: the time evolution of concurrence of qubits $(1,\,4)$ [*C*(*t*) in Eq. (11), solid green line] and [$C^{\prime }(t)$ in Eq. (11), dashed black line] versus the scaled time $\lambda t$ when the initial states of qubits pairs $(1,\,2)$, $(3,\,4)$ are as in Eq. (1). Right plot: the time evolution of concurrence of qubits $(1,\,8)$ [$C_4(t,\tau )$ in Eq. (21), solid green line] and [$C_{2}(t,\tau )$ in Eq. (21), dashed black line] versus the scaled time $\lambda \tau$ with $\lambda t=6$.

**Figure 3:**
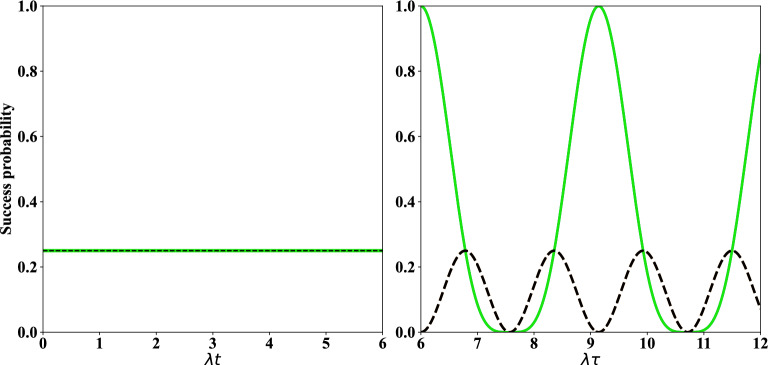
The time evolution of success probability of entangled state corresponding to qubits (1, 4) versus the scaled time $\lambda t$ (left plot), and qubits (1, 8) versus the scaled time $\lambda \tau$ (right plot). The details of the left and right plots are the same as Fig. 2.

In this way, we distinctly arrived at,

(i) For *i*=1, i.e., when the initial state is $|\psi (t)\rangle _{1,4}\otimes |\psi (t)\rangle _{5,8}$,
  14 $$\begin{aligned} \zeta _1(t) &=  \frac{L^2_2(t)}{P_{1,4}(t)},\qquad \eta _1(t)= \frac{L^2_3(t)}{P_{1,4}(t)},\qquad \beta _1(t)=\xi _1(t)= \frac{L_2(t)L_3(t)}{P_{1,4}(t)}, \end{aligned}$$
(ii) For *i*=2, i.e., when the initial state is $|\psi (t)\rangle _{1,4}\otimes |\psi ^{\prime }(t)\rangle _{5,8}$,
  15 $$\begin{aligned} \zeta _2(t) &=  \frac{L_1(t)L_2(t)}{\sqrt{P_{1,4}(t)P^{\prime }_{1,4}(t)}},\qquad \beta _2(t)=\frac{L_2(t)L_4(t)}{\sqrt{P_{1,4}(t)P^{\prime }_{1,4}(t)}},\\\nonumber \xi _2(t) &=  \frac{L_1(t)L_3(t)}{\sqrt{P_{1,4}(t)P^{\prime }_{1,4}(t)}},\qquad \eta _2(t)=\frac{L_3(t)L_4(t)}{\sqrt{P_{1,4}(t)P^{\prime }_{1,4}(t)}}, \end{aligned}$$
(iii) For *i*=3, i.e., when the initial state is $|\psi ^{\prime }(t)\rangle _{1,4}\otimes |\psi (t)\rangle _{5,8}$,
  16 $$\begin{aligned} \zeta _3(t) &=  \frac{L_1(t)L_2(t)}{\sqrt{P_{1,4}(t)P^{\prime }_{1,4}(t)}},\qquad \beta _3(t)=\frac{L_1(t)L_3(t)}{\sqrt{P_{1,4}(t)P^{\prime }_{1,4}(t)}},\nonumber \\ \xi _3(t) &=  \frac{L_2(t)L_4(t)}{\sqrt{P_{1,4}(t)P^{\prime }_{1,4}(t)}},\qquad \eta _3(t)=\frac{L_3(t)L_4(t)}{\sqrt{P_{1,4}(t)P^{\prime }_{1,4}(t)}}, \end{aligned}$$
(iv) For *i*=4, i.e., when the initial state is $|\psi ^{\prime }(t)\rangle _{1,4}\otimes |\psi ^{\prime }(t)\rangle _{5,8}$,
  17 $$\begin{aligned} \zeta _4(t)=\frac{L^2_1(t)}{P^{\prime }_{1,4}(t)},\qquad \eta _4(t)= \frac{L^2_4(t)}{P^{\prime }_{1,4}(t)},\qquad \beta _4(t)=\xi _4(t)= \frac{L_1(t)L_4(t)}{P^{\prime }_{1,4}(t)}. \end{aligned}$$
  By performing the final interaction between qubits $(4,\,5)$ introduced in (6), the time evolution of the state of qubits $(1,\,4,\,5,\,8)$ takes the form,
  18 $$\begin{aligned} |\psi _i(\tau )\rangle _{1,4,5,8} &=  \left( m_2^i(t,\tau )|e,g\rangle _{1,8}+m_3^i(t,\tau )|g,e\rangle _{1,8}\right) |e,g\rangle _{4,5} \nonumber \\{} & {} \quad +\left( m_1^i (t,\tau ) |e,g\rangle _{1,8}+m_4^i(t,\tau )|g,e\rangle _{1,8}\right) |g,e\rangle _{4,5}\nonumber \\{} & {} \quad + m_5^i(t,\tau )|e,e\rangle _{1,8}|g,g\rangle _{4,5} + m_6^i(t,\tau )|g,g\rangle _{1,8}|e,e\rangle _{4,5}, \end{aligned}$$
  where
  19 $$\begin{aligned} m_1^i (t,\tau ) &=  \zeta _i(t)\cos (\lambda \tau ),\qquad \qquad m_2^i (t,\tau )=-i \zeta _i(t)\sin (\lambda \tau ),\nonumber \\ m_3^i (t,\tau ) &=  \eta _i(t)\cos (\lambda \tau ),\qquad \qquad m_4^i (t,\tau )=-i \eta _i(t)\sin (\lambda \tau ),\nonumber \\ m_5^i (t,\tau ) &=  \beta _{i}(t),\qquad \qquad \qquad \qquad m_6^i (t,\tau )=\xi _{i}(t). \end{aligned}$$
  Via measuring the states $|e,g\rangle _{4,5}$ and $|g,e\rangle _{4,5}$ on the state (18), the normalized entangled states of the target SC qubits (1, 8) are respectively obtained as,
  20 $$\begin{aligned} |\psi _{i}(\tau )\rangle _{1,8} &=  \dfrac{1}{\sqrt{P_i(t,\tau )}} \left( m_2^i(t,\tau )|e,g\rangle +m_3^i(t,\tau )|g,e\rangle \right) _{1,8},\nonumber \\ |\psi _{i}^{\prime }(\tau )\rangle _{1,8} &=  \dfrac{1}{\sqrt{P^{'}_{i}(t,\tau )}} \left( m_1^i(t,\tau )|e,g\rangle +m_4^i(t,\tau )|g,e\rangle \right) _{1,8}, \end{aligned}$$
  where the associated concurrences and success probabilities respectively read as,
  21 $$\begin{aligned} C_i(t,\tau )=\dfrac{2|m_2^{i*} (t,\tau )m_3^i(t,\tau )|}{P_i(t,\tau )},\qquad \qquad C^{\prime }_{i}(t,\tau )=\dfrac{2|m_1^{i*} (t,\tau )m_4^i(t,\tau )|}{P^{'}_{i}(t,\tau )}, \end{aligned}$$
  and
  22 $$\begin{aligned} P_i(t,\tau )=|m_2^i(t,\tau )|^2+|m_3^i(t,\tau )|^2,\qquad \qquad P^{'}_i(t,\tau )=|m_1^i(t,\tau )|^2+|m_4^i(t,\tau )|^2. \end{aligned}$$
  The time evolution of concurrence and success probability of qubits $(1,\,4)$ and target qubits $(1,\,8)$ are respectively considered in Figs. 2 and 3. Notice that the time evaluation of concurrence and success probability of the SC qubits $(5,\,8)$ are similar to SC qubits $(1,\,4)$ in all figures in this paper. In the left plot of Fig. 2 the regular periodic behavior of concurrence is shown. From Fig. 2 one can see that the produced entangled states related to qubits $(1,\,4)$ and target qubits $(1,\,8)$ have been converted to some maximally entangled states at some moments of time. One can also observe that the success probability in the left plot of Fig. 3 is time-independent. In the right plot of Fig. 3, the acceptable maxima for success probability have been achieved.

We identify the total success probability of the protocol. The total success probabilities of generating the entangled states for qubits $(1, \,4)$ as well as $(5, \,8)$ are respectively achieved as,

23 $$\begin{aligned} P^{Tot}_{1,4}(t)= P_{1,4}(t)+P^{'}_{1,4}(t),\qquad \qquad P^{Tot}_{5,8}(t)= P^{Tot}_{1,4}(t), \end{aligned}$$

where $P_{1,4}(t), P^{'}_{1,4}(t)$ have been defined in (10). In fact, in term $P^{Tot}_{1,4}(t)$ ($P^{Tot}_{5,8}(t)$) in (23), achieving the entangled state of the pairs $(1, \,4)$ ($(5, \,8)$) is important, not which of the states $|\psi (t)\rangle _{1, 4}$ or $|\psi ^{\prime }(t)\rangle _{1, 4}$ ($|\psi (t)\rangle _{5, 8}$ or $|\psi ^{\prime }(t)\rangle _{5, 8}$) is obtained. The probability of success obtained after applying the measurement on qubits $(4, \,5)$, *i.e.*, the total success probability of achieving the entangled states for pair $(1, \,8)$ in this stage is equal to

24 $$\begin{aligned} P^{Tot}_{1,8}(t,\tau )=\sum _{i=1}^{4} P_{i}(t,\tau )+P^{'}_{i}(t,\tau ), \end{aligned}$$

where $P_{i}(t,\tau ), P^{'}_{i}(t,\tau )$ have been defined in (22) with $i={1,\,2,\,3,\,4}$. Similar to our explanations about Eq. (23), we state that, for $P^{Tot}_{1,8}(t,\tau )$ in Eq. (24), achieving the entangled states for pair $(1, \,8)$ is important, not which of the states $|\psi _i(\tau )\rangle _{1, 8}$ or $|\psi ^{\prime }_i(\tau )\rangle _{1, 8}$ are obtained. Therefore, the whole success probability of achieving the entangled states according to the given explanations is equal to

25 $$\begin{aligned} P(t,\tau )=P^{Tot}_{1,4}(t)\times P^{Tot}_{5,8}(t)\times P^{Tot}_{1,8}(t,\tau ). \end{aligned}$$

**Figure 4:**
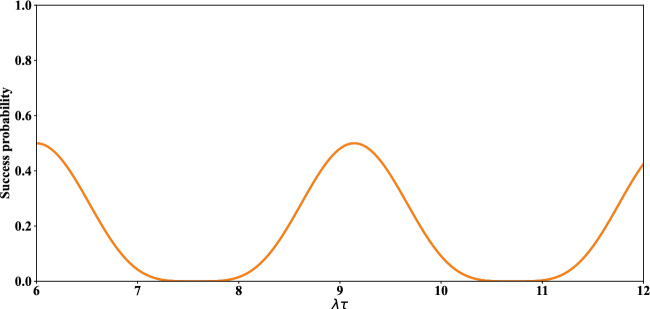
The time evolution of whole success probability of entangled states of qubits $(1, \,8)$ versus the scaled time $\lambda \tau$ with $\lambda t=6$.

In Fig. 4, the whole success probability of this protocol in the absence of dissipation, $P(t,\tau )$, has been plotted. It is observed that this quantity is periodic, while acceptable value of maximum success probability (and enough interval of times around that) has been achieved.

In the next section, the above-mentioned process for designing the entanglement swapping protocol based on SC qubits is considered in the presence of dissipation.

## Dissipative entanglement swapping based on SC qubits

The considered system in the above section studied in the ideal condition (in the absence of dissipation sources). To take into account real physical situation, the relaxation rate $(\Gamma _i)$ and the pure dephasing rate $(\gamma _i)$ of the *i*th qubit are also considered. The time evolution of the system including qubits 1, 2, 3, 4 (or qubits 5, 6, 7, 8) shown by $\rho _{1-4}(t)$ with relaxation and pure dephasing rates of qubits should be investigated via the master equation. Another phenomenon that is important in studying SC qubits in open quantum systems is quantum jump. In fact, an open quantum system can be studied using the Lindblad equation, consisting of a Hermitian Hamiltonian part and a non-Hermitian one. The non-Hermitian Hamiltonian can be divided into two parts: quantum jumps, and a term that yields coherent non-unitary evolution. The instantaneous switching between energy levels are created by quantum jumps. Also, a term can be known as a quantum jump because in a quantum trajectory approach this term is responsible for the abrupt stochastic change of the wavefunction^72^. Moreover, the effects of quantum jumps can be eliminated for instance through post-selection^73,74^:

26 $$\begin{aligned} \frac{d \rho _{1-4}(t)}{dt}=-i[H_{\textrm{eff}},\rho _{1-4}(t)] -\sum _{i=2,3}\dfrac{\Gamma _i}{2} D\left[ \sigma ^{-}_i\right] -\sum _{i=2,3}\dfrac{\gamma _i}{2} D\left[ \sigma ^{z}_i\right] , \end{aligned}$$

where

27 $$\begin{aligned} D[A] &=  (n_{th}+1) \left( 2 A\rho _{1-4}(t) A^{\dagger }-\rho _{1-4}(t) A^{\dagger } A-A^{\dagger } A\rho _{1-4}(t) \right) \nonumber \\{} & {} \quad + n_{th}\left( 2 A^{\dagger }\rho _{1-4}(t) A-\rho _{1-4}(t) A A^{\dagger }-AA^{\dagger } \rho _{1-4}(t) \right) , \end{aligned}$$

is the Lindblad operator^74^. Now, for simplicity and without loss of generality we assume that $\Gamma _2=\Gamma _3=\Gamma$, $\gamma _2=\gamma _3=\gamma$ and finally we set $\gamma =\Gamma$^75^. It should be noted that the effects of different $\gamma$’s and $\Gamma$’s on the time evolution of the considered parameters like concurrence and the whole success probability of this protocol are considered. The results showed that the general behavior of concurrence, the whole success probability and fidelity of the protocol do not essentially change by taking into account different values of the two mentioned parameters. We would like to mention that according to the definitions of dissipation rates $\Gamma$ and $\gamma$ in the present work and also based on the definition of $T_1$ and $T_2$ which are respectively known as relaxation and dephasing times of transmon qubits^76–78^, it is clear that $\Gamma =1/T_1$ and $\gamma =1/T_2$^79^. In our numerical analysis, we have considered the coupling strength to be much greater than the decay and dephasing rates^35^. After applying the projection (measurement) operators $|e,g\rangle _{2,3}\langle e,g|$ and $|g,e\rangle _{2,3}\langle g,e|$ on $\rho _{1-4}(t)$, the concurrences of qubits $(1,\,4)$ (or qubits $(5,\,8)$) and their corresponding success probabilities may be obtained, numerically. Finally, to consider the effects of relaxation and pure dephasing rates of transmon qubits on the distributed entangled state of target qubits $(1,\,8)$ the following master equation for qubits $(1,\,4,\,5,\,8)$ is numerically solved,

28 $$\begin{aligned} \frac{d\rho _{1,4,5,8}(\tau )}{d\tau }=-i[H_{\textrm{eff}},\rho _{1,4,5,8}(\tau )] - \sum _{i=4,5}\dfrac{\Gamma _i}{2} D\left[ \sigma ^{-}_i\right] - \sum _{i=4,5}\dfrac{\gamma _i}{2} D\left[ \sigma ^{z}_i\right] , \end{aligned}$$

where $D\left[ A\right]$ has been defined in Eq. (27), however clearly now with $\rho _{1,4,5,8}(\tau )$, while $\rho _{1,4,5,8}$ is the time evolution of the system including qubits 1, 4, 5, 8 with relaxation and pure dephasing rates of qubits. The distributed entangled state of qubits $(1,\,8)$ is then readily achieved after applying proper measurements, by applying the projection operators $|e,g\rangle _{4,5}\langle e,g|$ and $|g,e\rangle _{4,5}\langle g,e|$ on $\rho _{1,4,5,8}(\tau )$. To study the time evolution of final entanglement between qubits $(1,\,8)$, the concurrence is numerically calculated.

**Figure 5:**
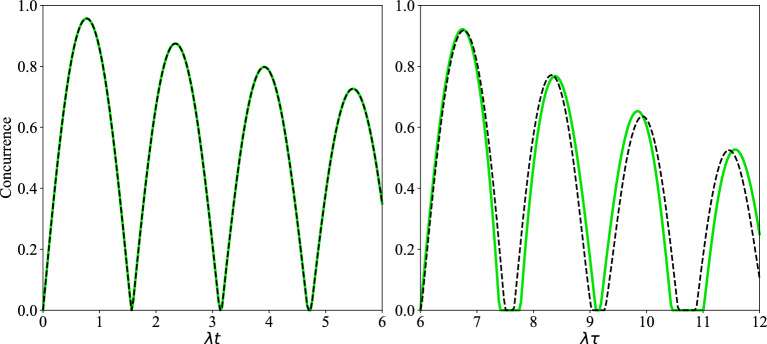
The time evolution of concurrence: versus the scaled time $\lambda t$ (left plot), the scaled time $\lambda \tau$ (right plot). The details of the left and right plots are the same as Fig. 2 with n$_{th}$=0.25, $\Gamma =\gamma =0.01 \lambda$.

**Figure 6:**
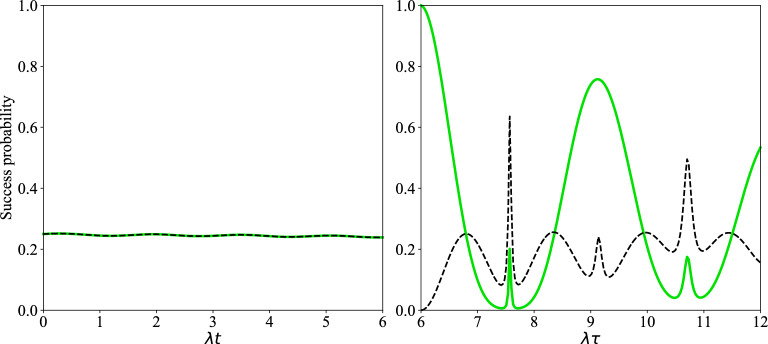
The time evolution of success probability of entangled state related qubits (1, 4) versus the scaled time $\lambda t$ (left plot), qubits (1, 8) versus the scaled time $\lambda \tau$ (right plot). The details of the left and right plots are the same as Fig. 2 with n$_{th}$=0.25, $\Gamma =\gamma =0.01 \lambda$.

Now, we pay our attention to the time evolution of concurrence and success probability of the distributed entangled states in the presence of thermal noise respectively considered in Figs. 5 and 6. In the left plot of Figs. 5 and 6 the acceptable amounts of concurrence (success probability) of the produced entangled state of qubits $(1,\,4)$ and $(5,\,8)$ are observed. Also, in these plots the behavior of two green and black curves is similar. In the left plot of Fig. 5, it is obviously observed that the maxima of concurrence of entangled states of qubits $(1,\,4)$ and $(5,\,8)$ are decreased as time goes on. In the right plot of Figs. 5 and 6 the concurrences (success probabilities) of distributed entangled states of target qubits $(1,\,8)$ are considered. In the right plot of Fig. 5 the maximum of concurrence is decreased with time. Also, the two curves of this figure move apart with time. In addition, the time interval of death of entanglement in dashed black curve is increased with time, but the behavior of solid green line in this interval is irregular. In the right plot of Fig. 6 the origin of two curves is completely different, in fact the solid green curve is started from 1 but the dashed black curve is started from zero. But, in these curves the values of maxima of success probability of obtaining the entangled states for qubits (1,8) are acceptable.

**Figure 7:**
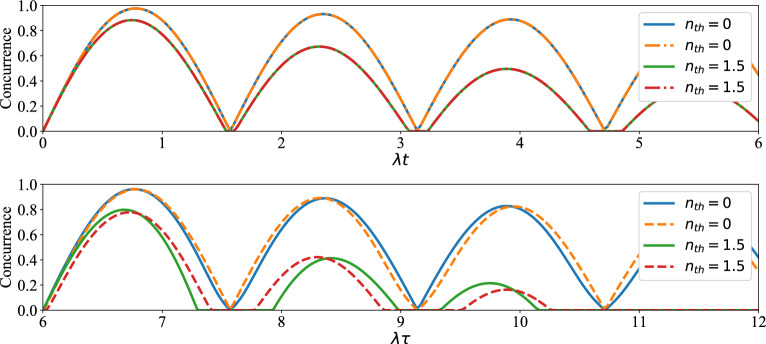
Up plot: The time evolution of concurrence of qubits $(1,\,4)$ versus the scaled time $\lambda t$ after measuring the qubits $(2,\,3)$ if results in $|e,g\rangle$ (solid lines) and $|g,e\rangle$ (dashed lines) when the initial states of qubit pairs $(1,\,2)$, $(3,\,4)$ are prepared as in Eq. (1) of manuscript. Down plot: The time evolution of concurrence of qubits $(1,\,8)$ versus the scaled time $\lambda \tau$ after measuring $|g,e\rangle$ on qubits $(4,\,5)$ when the initial states of qubits $(1,\,4)$ and $(5,\,8)$ at $\lambda t$ have been respectively measured as $|g,e\rangle _{2,3}$, $|g,e\rangle _{6,7}$ (solid lines) and $|e,g\rangle _{2,3}$, $|g,e\rangle _{6,7}$ (dashed lines) with $\lambda t=6$ and $\Gamma =0.01 \lambda$.

**Figure 8:**
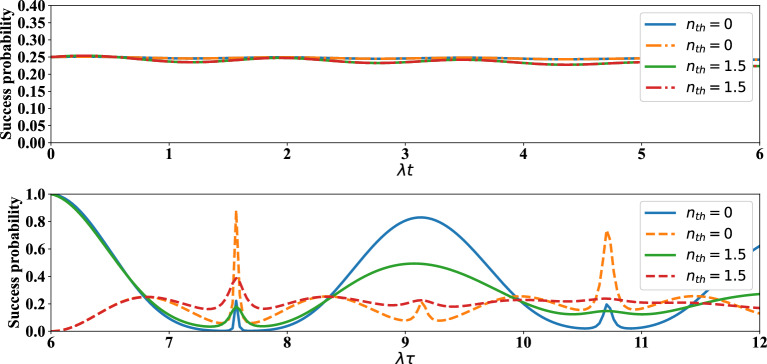
The time evolution of success probability of entangled state related qubits (1, 4) versus the scaled time $\lambda t$ (up plot), qubits (1, 8) versus the scaled time $\lambda \tau$ (down plot). The details of the up and down plots are the same as Fig. 7.

Finally, let us examine the effect of average number of thermal photons on the time evolution of concurrence and success probability of the distributed entangled states of qubits (1, 4), (5, 8) and (1, 8). As can be seen from Fig. 7, the time interval in which death of entanglement occurs is increased via increasing $n_{th}$. Also, in this figure, it can be easily found that, the maxima of the concurrence are greatly decreased via increasing the average number of thermal photons. From up plot of Fig. 8 it is visible that the success probability of obtaining entangled state for qubits (1,4) or (5,8) is almost equal to 0.25. In fact, the behavior of success probability of entangled state corresponding to qubits (1,4) in this plot is almost time-independent and similar for different conditions, but in the down plot of this figure one can see the irregular behavior of success probability of entangled state associated with qubits (1,8). In down plot of Fig. 8, some maxima of success probability for $n_{th}=0$, $n_{th}=1.5$ are acceptable. The entangled states of qubits (1,4) in Fig. 8 are indeed the states that have been obtained after applying the projection operators $|e,g\rangle _{2,3}\langle e,g|$ and $|g,e\rangle _{2,3}\langle g,e|$ on $\rho _{1-4}(t)$ in Eq. (26).

To ensure that the approximation for obtaining the effective Hamiltonian (6) is reasonable, master equations (26) and (28) were also solved numerically by considering $H_I$ introduced in Eq. (4) instead of $H_{\textrm{eff}}$ in (6). It was observed that the plots of success probability and concurrence, i.e., Figs. 6 and 7, experience no noticeable change.

**Figure 9:**
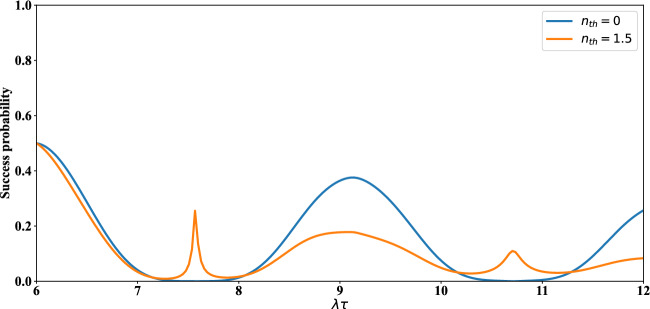
The time evolution of whole success probability of entangled states of qubits $(1, \,8)$ versus the scaled time $\lambda \tau$ with $\lambda t=6$ and $\Gamma =\gamma =0.01 \lambda$.

Finally, the whole success probability of the protocol in the presence of dissipation is achieved by repeating the calculations from Eqs. (23) to (25), but by considering the $\rho _{1-4}(t)$ and $\rho _{1,4,5,8}(\tau )$ identified respectively in (26) and (28). In Fig. 9, the whole success probability of this protocol, $P(t,\tau )$, in the presence of dissipation has been plotted. The maxima of success probability with $n_{th}=0$ (blue line) are about 0.5, 0.4 and 0.3, while these maxima with $n_{th}\ne 0$ (orange line) have been decreased. It should be noted that, these maximum values as well as the adjacent values in some finite intervals of time (even in the presence of thermal dissipation) are satisfactorily acceptable in a reliable entanglement swapping scheme (see Refs.^11,80,81^).

**Figure 10:**
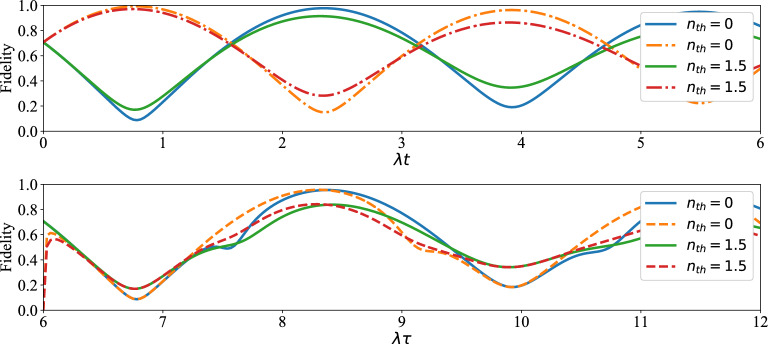
Up plot: the time evolution of fidelity of entangled qubits $(1,\,4)$ and Bell state (1) versus the scaled time $\lambda t$ after measuring $|e,g\rangle$ (solid curves) and $|g,e\rangle$ (dashed curves) on qubits $(2,\,3)$ when the initial states of qubits pairs $(1,\,2)$, $(3,\,4)$ are as in Eq. (1). Down plot: the time evolution of fidelity of entangled qubits (1, 8) and Bell state (1) versus the scaled time $\lambda \tau$ after measuring $|g,e\rangle$ on qubits $(4,\,5)$ when the initial states of qubits $(1,\,4)$ and $(5,\,8)$ at $\lambda t$ have been obtained respectively by measuring $|g,e\rangle _{2,3}$, $|g,e\rangle _{6,7}$ (solid curves) and $|e,g\rangle _{2,3}$, $|g,e\rangle _{6,7}$ (dashed curves) with $\lambda t=6$ and $\Gamma =\gamma =0.01 \lambda$.

At last, due to the importance of calculating the fidelity of the produced or distributed entangled states in entanglement swapping protocols, this measure was considered to study the closeness of the achieved entangled states for qubits $(1, \,4)$, $(1, \,8)$ to the initial Bell state (1). As shown in Fig. 10, satisfactory amount of fidelity was achieved for the produced entangled states. Also, it can be easily seen that the maxima of fidelity are decreased via increasing the average number of thermal photons. In fact, thermal photons have a destructive role on the maxima of fidelity.

## Summary and conclusions

In this paper, we studied the production of distributed entangled state of SC qubits in the absence and presence of dissipation using entanglement swapping protocol in three successive stages. In our protocol eight SC qubits $(1,2,\ldots ,8)$ have been considered where the pairs $(i,\,i+1$ with $i=1,\,3,\,5,\,7)$ have already prepared in maximally entangled states. The entangled states of SC qubits $(1,\,4)$ and $(5,\,8)$ have been achieved by implementing external magnetic fields on capacitively coupled pairs $(2,\,3)$ and $(6,\,7)$ and operating proper measurements. Finally, by applying external magnetic fields on qubits $(4,\,5)$ and via operating suitable measurements, the target SC qubits $(1,\,8)$ converted to entangled state. In the absence of dissipation the produced entangled states for qubits $(1,\,4)$ or $(5,\,8)$ as well as the target qubits $(1,\,8)$ converted to maximally entangled states at some moments of time. We observed that the maximum of success probability of entangled state production reaches to 1 in some particular conditions. Moreover, we have also studied the entanglement swapping in the presence of dissipation, considered via relaxation rates of SC qubits, as well as the thermal noise effects. We found that the maxima of concurrence and success probability are decreased as time goes on in the presence of relaxation rate and via increasing the average number of thermal photons. At last, the whole success probability and the fidelity with satisfactory amounts have been achieved for the desired distributed entangled states.

### Supplementary Information

Supplementary Information.

## Data Availability

All data generated or analysed during this study are included in this published article.
