# Supplementary material for: Multistage entanglement swapping using superconducting qubits in the absence and presence of dissipative environment without Bell state measurement
Source: Sci Rep. 2023 Sep 28;13:16342. doi: 10.1038/s41598-023-43592-y (PMC10539405; doi:10.1038/s41598-023-43592-y)
Supplement: Supplementary file 1 — Supplementary Information. [file 41598_2023_43592_MOESM1_ESM.pdf]

## Appendix A

We propose an algorithm composed of gates from some standard universal gate set to prepare the entangled states. The initial state of four SC qubits (1, 2, 3, 4) is considered as $|\psi\rangle_{1,2} \otimes |\psi\rangle_{3,4}$, where the pairs (1, 2) and (3, 4) have been prepared in the following entangled state:

$$|\psi\rangle_{i,i+1} = \frac{1}{\sqrt{2}} \left(|e,g\rangle + |g,e\rangle\right)_{i,i+1}, \quad i = 1, 3. \tag{1}$$

At first, a Hadamard gate is applied on SC qubit 1, so the normalized state of the SC qubits (1, 2, 3, 4) takes the form,

$$\begin{aligned} |\psi\rangle^{'}_{(1-4)} &= \frac{1}{2\sqrt{2}} \Big( |g,g\rangle_{1,4} |g,e\rangle_{2,3} - |e,g\rangle_{1,4} |g,e\rangle_{2,3} \\ &+ |g,e\rangle_{1,4} |g,g\rangle_{2,3} - |e,e\rangle_{1,4} |g,g\rangle_{2,3} + |g,g\rangle_{1,4} |e,e\rangle_{2,3} \\ &+ |e,g\rangle_{1,4} |e,e\rangle_{2,3} + |g,e\rangle_{1,4} |e,g\rangle_{2,3} + |e,e\rangle_{1,4} |e,g\rangle_{2,3} \Big). \end{aligned} \tag{2}$$

In the next step, the state (2), via applying a Pauli-X gate on SC qubit 4, is converted to the following state:

$$\begin{aligned} |\psi\rangle^{''}_{(1-4)} &= \frac{1}{2\sqrt{2}} \Big( |g,e\rangle_{1,4} |g,e\rangle_{2,3} - |e,e\rangle_{1,4} |g,e\rangle_{2,3} \\ &+ |g,g\rangle_{1,4} |g,g\rangle_{2,3} - |e,g\rangle_{1,4} |g,g\rangle_{2,3} + |g,e\rangle_{1,4} |e,e\rangle_{2,3} \\ &+ |e,e\rangle_{1,4} |e,e\rangle_{2,3} + |g,g\rangle_{1,4} |e,g\rangle_{2,3} + |e,g\rangle_{1,4} |e,g\rangle_{2,3} \Big). \end{aligned} \tag{3}$$

In the last step, a CNOT gate (See Appendix B to get information about implementing CNOT gate.) is applied to the flip SC qubit 4 conditioned on SC qubit 1, and the following state is obtained,

$$\begin{aligned} |\psi\rangle^{'''}_{(1-4)} &= \frac{1}{2\sqrt{2}} \Big( |g,e\rangle_{1,4} |g,e\rangle_{2,3} - |e,g\rangle_{1,4} |g,e\rangle_{2,3} \\ &+ |g,g\rangle_{1,4} |g,g\rangle_{2,3} - |e,e\rangle_{1,4} |g,g\rangle_{2,3} + |g,e\rangle_{1,4} |e,e\rangle_{2,3} \\ &+ |e,g\rangle_{1,4} |e,e\rangle_{2,3} + |g,g\rangle_{1,4} |e,g\rangle_{2,3} + |e,e\rangle_{1,4} |e,g\rangle_{2,3} \Big). \end{aligned} \tag{4}$$

At this stage, if the result of measurement on SC qubits (2, 3) is $|g,e\rangle_{2,3}$ or $|e,e\rangle_{2,3}$, the state of the qubits (1, 4) is respectively converted to the following Bell states:

$$|\psi\rangle_f = \frac{1}{\sqrt{2}} \left(|g,e\rangle - |e,g\rangle\right)_{(1,4)}, \qquad |\psi\rangle^{'}_f = \frac{1}{\sqrt{2}} \left(|g,e\rangle + |e,g\rangle\right)_{(1,4)}. \tag{5}$$

Finally, the desired entangled state is achieved via applying a Pauli-Z gate on SC qubit 1 on $|\psi\rangle_f$. It is observed that the desired entangled state of SC qubits (1, 4) is obtained via applying universal gates.

## Appendix B

Any single-qubit operation can be implemented by applying the appropriate time-dependent electromagnetic field which resonantly interacts with the selected qubit and can be derived from [1, 2, 3],

$$U(\theta,\phi) = \begin{pmatrix} \cos\theta & e^{-i\phi}\sin\theta \\ e^{i\phi}\sin\theta & -\cos\theta \end{pmatrix}, \tag{6}$$

where $\theta = 2|\lambda|t$, in which $\lambda$ is the Rabi frequency of the qubit and $\phi$ and $t$ are respectively the phase and the time duration of the applied electromagnetic field. As a result, the Hadamard gate can be implemented through Eq. (6) by setting the applied time-dependent electromagnetic field such that $\theta = \pi/4$.
In this line, C-NOT quantum gate on a pair of SC qubit can be achieved via applying microwave pulses with appropriate frequency [4, 5] via gate matrix,

$$\hat{R}_{1_C 0_T - 1_C 1_T} = \begin{pmatrix} 1 & 0 & 0 & 0 \\ 0 & 1 & 0 & 0 \\ 0 & 0 & \cos(\frac{\omega\tau}{2}) & i\sin(\frac{\omega\tau}{2}) \\ 0 & 0 & i\sin(\frac{\omega\tau}{2}) & \cos(\frac{\omega\tau}{2}) \end{pmatrix}, \tag{7}$$

where $\tau$ and $\omega$ are the pulse length and the Rabi frequency of qubit, respectively. Also, the subscripts in $\hat{R}$ represent the control (C) qubit and the target (T) qubit. This rotation performs the ideal C-NOT gate if one sets $\omega\tau = \pi$.

## References


## References



[1] Y.-x. Liu, L. Wei, J. Tsai, and F. Nori, Phys. Rev. Lett. **96**, 067003 (2006).

[2] G. Wendin, Rep. Prog. Phys. **80**, 106001 (2017).

[3] S. Danilin, A. Vepsäläinen, and G. S. Paraoanu, Phys. Scr. **93**, 055101 (2018).

[4] J. Plantenberg, P. De Groot, C. Harmans, and J. Mooij, Nature **447**, 836 (2007).

[5] P. De Groot, S. Ashhab, A. Lupaşcu, L. DiCarlo, F. Nori, C. Harmans, and J. Mooij, New J. Phys. **14**, 073038 (2012).
